# First description of co-occurrence of 49,XXXXY and X-linked Cornelia de Lange syndrome: case report

**DOI:** 10.3389/fendo.2025.1688852

**Published:** 2025-10-17

**Authors:** Sumaiya Al-Rashdi, Lori Ekstrom, Aedin Collins, Heidi L. Schulz, April Dinwiddie, Carole Samango-Sprouse, Margaret Olaya, Elizabeth Moser, Andrew J. Green

**Affiliations:** ^1^ Department of Clinical Genetics, Children's Health Ireland at Crumlin, Dublin, Ireland; ^2^ Department of Paediatrics, Children's Health Ireland at Crumlin, Dublin, Ireland; ^3^ Zentrum für Humangenetik Tübingen, Tübingen, Germany; ^4^ Department of Research, The Focus Foundation, Davidsonville, MD, United States; ^5^ School of Medicine and Health Sciences, George Washington University, Washington, DC, United States; ^6^ Florida International University, Department of Human and Molecular Genetics, Miami, FL, United States; ^7^ School of Medicine and Medical Science, University College Dublin, Dublin, Ireland

**Keywords:** 49,XXXXY, SMC1A Cornelia de Lange syndrome, epigenetic signature, X-linked disorders, case report

## Abstract

49,XXXXY is a rare sex chromosome aneuploidy with an estimated incidence of 1 in 85,000–100,000 newborn males. Individuals with this syndrome exhibit variable clinical manifestations, typically including developmental delay, intellectual deficits, hypogonadism, and distinctive facial features such as ocular hypertelorism, epicanthic folds, a flat nasal bridge, prognathism, folded-over ears, and a short neck. Unlike patients with Klinefelter syndrome, they are often short in stature. Cornelia de Lange syndrome (CdLS) is an unrelated rare disorder with an incidence of 1 in 10,000–30,000 live births, affecting both sexes. CdLS shares overlapping features with 49,XXXXY, including intellectual deficits and hypogonadism. However, it also presents with unique facial characteristics, such as synophrys, thick or highly arched eyebrows, low-set ears, upturned nasal tips, long eyelashes, and microcephaly. CdLS is a clinically and genetically heterogeneous condition, with severe cases involving congenital malformations including limb anomalies, and milder cases showing only subtle facial dysmorphism. Both syndromes may also involve cardiac and renal anomalies. We report the first documented concurrence of 49,XXXXY and X-linked CdLS, emphasizing the challenges in diagnosis and the phenotypic overlap between these two rare syndromes, and propose a theoretical mechanism for the co-occurrence.

## Introduction

49,XXXXY syndrome is a rare chromosomal aneuploidy with an incidence of approximately 1 in 85,000–100,000 male births. It is characterized by moderate to severe intellectual disability, hypogonadism, facial dysmorphism, and skeletal anomalies. Cornelia de Lange syndrome (CdLS) is a distinct rare genetic condition with a prevalence of 1 in 10,000–30,000 live births. Most cases (80%) are sporadic autosomal dominant events caused by pathogenic variants in the NIPBL gene. However, rare cases can occur in the X-linked genes SMC1A and HDAC8. Clinical manifestations of CdLS include distinctive facial abnormalities, limb and cardiac malformations, growth retardation, and intellectual disability.

The co-occurrence of these two syndromes has not been previously reported in the literature, making this case unique.

## Clinical details

A first child to unrelated parents was born at 37 weeks gestation with a birth weight of 2.57 kg (50^th^ centile). He experienced hypoglycemia shortly after birth but did not require neonatal unit admission. He appeared well initially but at 3 months of age suffered a severe respiratory syncytial virus (RSV) infection requiring extracorporeal membrane oxygenation (ECMO) support. During his pediatric intensive care unit (PICU) stay, he was found to have atrial and ventricular septal defects (ASD and VSD), a left undescended testis and a concealed penis suggesting hypogonadism, and bilateral moderate sensorineural hearing loss. Neurologically, he displayed truncal hypotonia but otherwise had a normal neurological examination. He subsequently had successful surgical repair of his septal defects.

Both his parents were of normal stature, had normal childhoods, and had no developmental concerns.

By 18 months, the patient showed significant developmental delay. He was a bottom-shuffler, required assistance to drink from a bottle, and communicated using limited babbling and a few words. Clinically, he exhibited clinical and facial features suggestive of CdLS, including synophrys, a relatively long philtrum, small feet, tapered fingers, and fifth-finger clinodactyly (photographs not available). His weight, height and head circumference were on the 25^th^-50^th^ centile.

Genetic testing was pursued due to the combination of congenital anomalies and developmental concerns.

## Genetic testing results

A 60k oligonucleotide array analysis of peripheral blood DNA showed the presence of 2–3 extra copies of the X chromosome. Confirmatory peripheral blood G-banding karyotype analysis of 30 metaphases showed a 49,XXXXY karyotype in all cells.

**Figure 1:**
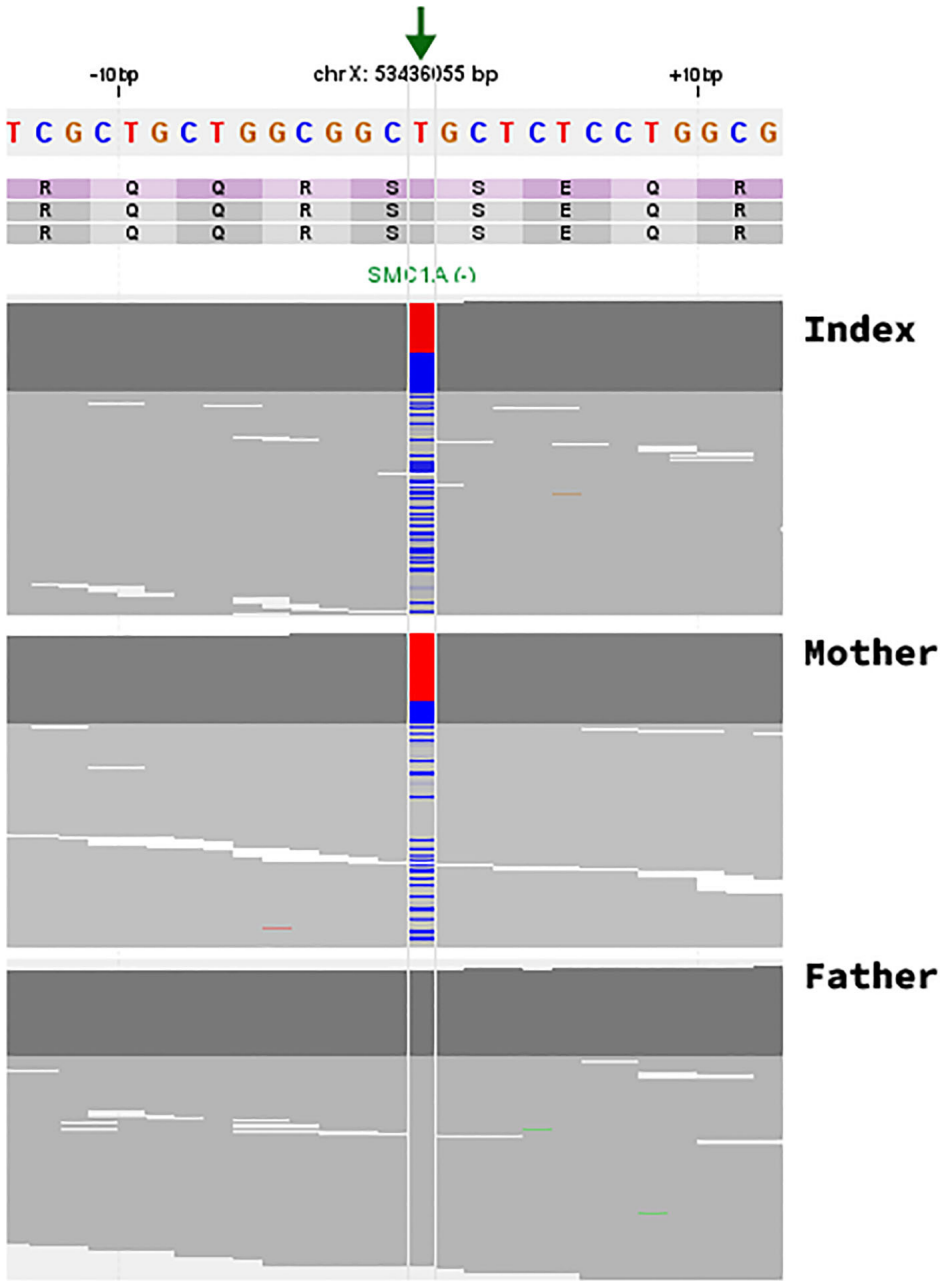
Identification of the c.1483A>G; p.Ser495Gly missense variant in the *SMC1A* gene. Visualization of NGS paired-end reads in an IGV-like genome browser. The arrow indicates the position (chrX:53436055 T>C (hg19), NM_006306.4) of the single nucleotide variation in the *SMC1A* gene in the index (122/226 reads; 45,86%, heterozygous), the mother (42/173 reads; 24.28%, mosaic), and the father (wildtype/variant not present). Note that the *SMC1A* gene is transcribed in the reverse direction and that the depicted genomic sequence is of the forward strand with the reference base (T) in red and the alternative allele (C) in blue.

Considering his dysmorphism and malformations, further investigation was carried out. Trio exome analysis of peripheral blood DNA by next generation sequencing detected a heterozygous variant in the X-linked SMC1A gene, c.1483A>G p.Ser495Gly NM_006306.4. The variant was present in a mosaic state in his mother’s blood with a variant allele frequency of 24% (**Figure 1**).

The variant was absent from the gnomAD global population set (ACMG PM2 moderate); there are fewer than expected missense variants in the *SMC1A* gene in the normal population, suggesting poor tolerance of missense variation (ACMG PP2 supporting); the variant was present in a mosaic state in his mother (ACMG PS2 moderate), and the phenotype is specific for a disease with a specific aetiology (ACMG PP4 Moderate). The variant is therefore classified as likely pathogenic by ACMG criteria (1).

For the index case, the allele frequency for the *SMC1A* variant was 0.46 over 266 reads and was therefore considered to be present in 2 of his 4 X chromosomes.

Single Nucleotide Polymorphism (SNP) analysis using Xp and Xq markers showed that all 4 of his X chromosomes were of maternal origin. In addition, he had two copies of each maternal chromosome, from which the assumption was made that the *SMC1A* variant was present on two identical X chromosomes from his mother.

Epigenetic methylation profiling was carried out on his blood DNA, using Episign methylation array analysis. This showed with moderate confidence an epigenetic signature characteristic of types 1–4 Cornelia de Lange syndrome.

## Discussion

We present a boy with clinical and genetic features of both 49,XXXXY syndrome and X-linked Cornelia de Lange syndrome.

49,XXXXY syndrome was first described in 1960s by Fraccaro et al. (2). It is considered a high grade aneuploidy with an incidence of 1 in 85–000 male births (3). Initially it was considered to be a variant of Klinefelter syndrome (KS). Both 49,XXXXY and 47, XXY (KS) share similar phenotypes and pathophysiology. They occur due to nondisjunction of the X chromosome usually during maternal meiosis I & II (4, 5). Generally, the incidence of sex chromosome aneuploidy increases with advanced maternal age, however there is no evidence that 49,XXXXY syndrome is associated with increased maternal age (6).

Classical 49,XXXXY syndrome has a triad of symptoms; intellectual disability, hypogonadism and radioulnar synostosis. 49,XXXXY syndrome is recognized to be a distinct entity from KS as affected males have more severe manifestations compared to KS (6). Affected males are characterized by learning difficulties and language delay. They may also have hypotonia with musculoskeletal manifestations. This phenotype was suggested to be due to firstly increase dosage of active genes in regions within the additional X chromosomes which escape X inactivation, and secondly asynchronous replication of the extra X chromosomes (7).

The only other reported case of a child with both a chromosome disorder and Cornelia de Lange was of a girl with mosaic Turner syndrome and NIPBL associated CdLS (8).

In this case the infant had features consistent with 49,XXXXY. He had infantile hypotonia, genital anomalies and congenital heart disease. The first line 60K oligonucleotide array on peripheral blood DNA indicated that there were additional signals from probes representing a significant copy number gain of all of the X chromosome, however it was not possible to give an accurate estimate of the exact copy number. Accurate copy number was provided by G Banded karyotyping confirmed by G band karyotyping to be 49,XXXXY. The proband therefore had both clinical and cytogenetic features of 49,XXXXY.

Whole exome sequencing did identify extra copies of the whole X chromosome, previously seen in both microarray and G-banded karyotype analysis. In addition, trio whole exome showed a likely pathogenic variant in the X chromosome gene *SMC1A*; c.1483A>G; p.Ser495Gly het. The same variant was found in a mosaic form in his mother (42/173 reads; 24.28%). *SMC1A* is a gene in which pathogenic alterations are seen in about 5% of patients with Cornelia de Lange Syndrome (CdLS) (9).

An epigenetic methylation signature for 4 types of CdLS has been characterized (10). The patient had this test carried out, which showed the presence of a CdLS methylation profile with moderate confidence. The moderate level of confidence in the result may be due to the presence of 2 normal X chromosomes, as well as the presence of 2 X chromosomes with the SMC1A variant. The presence of a characteristic phenotype of CdLS, the fact that the variant was present in a mosaic state in his mother, and the methylation profile result allowed the variant to be classified finally as likely pathogenic.

CdLS also has a spectrum of findings from mild to severe. The classic type (severe) is characterized by distinctive facial features, growth restriction, hypertrichosis, and upper-limb reduction defects. Unique facial features including synophrys, highly arched and/or thick eyebrows, long eyelashes, short nasal bridge with anteverted nares, small widely spaced teeth, and microcephaly. The milder forms can only show facial features consistent with the syndrome (9). The patient we present has typical facial features of CdLS, along with small hands and feet, tapering fingers, and 5^th^ finger clinodactyly (photographs not available).

CdLS has significant genetic heterogeneity. The most common cause is the presence of heterozygous loss of function variants in the autosomal gene *NIPBL* (80% of cases), *RAD21* (<1%), *SMC3* (1-2%) or *BRD4* (<1%). Rarely, the condition can show X linked dominant inheritance, due to hemizygous or heterozygous pathogenic variants in *HDAC8* (4%) or *SMC1A* (5%). There are significant genotype-phenotype correlations. Patients with loss of function variants in NIPBL are more likely to show the classic form of the condition with a higher frequency of congenital malformations, including limb defects (9). In contrast males and females with *SMC1A* CdLS have fewer malformations and less growth restriction that NIPBL related CdLS. Some 46,XY males with *SMC1A* variants have been reported to have very mild phenotypes (11).

The SMC1A protein forms a homodimer, which in turn interacts with a dimerised form of another CdLS gene, SMC3. SMC1 and SMC3 are proteins involved in the cohesin complex, and CdLS is often classified as a cohesinopathy. Cohesin complexes are involved in ensuring sister chromatids remain connected during mitosis until the chromatids separate in anaphase in human cells (12). There is also evidence from *C. elegans* that cohesin proteins are also involved in regulating meiotic division as well as mitotic division (13).

The *SMC1A* gene on Xp11.2 is reported to escape X inactivation. *SMC1A*-related CdLS is not due to altered levels of the *SMC1A* transcript, but rather that the mutant protein, expressed from one X chromosome in females, interferes with the function of the normal protein expressed from the other X chromosome, and thus exert a dominant negative effect in females on the dimerised SMC1 protein (10). As the male patient we describe has 4 X chromosomes, it is likely that he expresses the SMC1A protein containing the missense variant from two of those chromosomes, and expresses the normal SMC1A protein from the other 2 X chromosomes. Thus, the mechanism for his CdLS is similar to that seen in heterozygous females with *SMC1A* CdLS, rather than that seen in hemizygous males with *SMC1A* CdLS.

As the asymptomatic mother is mosaic for the *SMC1A* variant in both blood and oocytes, the variant is likely to have occurred as an early post-zygotic mitotic event on one of her X chromosomes. Subsequent to that mitotic event, the additional X chromosomes would have occurred due to two separate meiotic events, the first being non-disjunction of the X chromosome in meiosis I, followed by further non-disjunction of both X chromosomes in meiosis II. The resultant oocyte would then have 4 X chromosomes, 2 from each of the mother’s X chromosomes, 2 of which had the *SMC1A* variant. Given that there is evidence from *C. elegans* that cohesin complexes are involved in both mitotic and meiotic chromosome separation, one could speculate that the presence of a pathogenic SMC1A variant in the mother may have disrupted normal cohesin complex function leading to abnormal chromosome separation during both meiosis I and meiosis II, giving rise to 49,XXXXY. However, such speculation would need to be sustained by functional studies of *SMC1A* deficient human cells.

**Figure 2:**
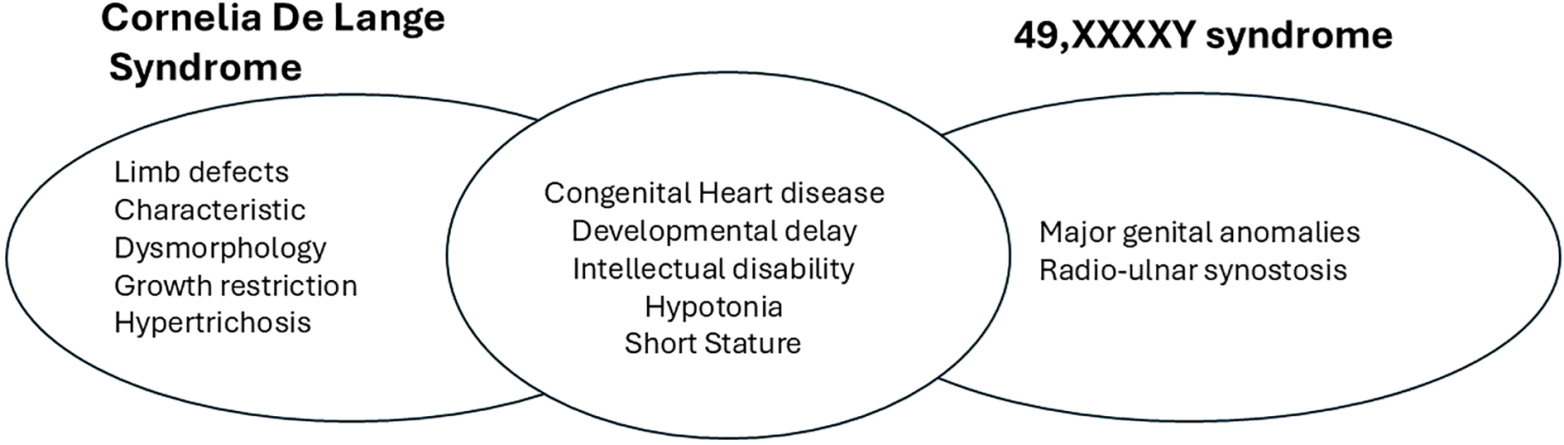
Comparison of clinical features of CdLS and 49,XXXXY.

The significant clinical problems in this child, namely hypotonia, hypogonadism, developmental delay, and congenital heart disease could be explained individually by either 49,XXXXY or by CdLS. However, it is likely that these clinical problems are caused by an additive combination of both conditions. For example, cardiac defects are seen in 14-20% of patients with 49,XXXXY (8), and 30% of patients with CdLS, although less common in *SMC1A* associated CdLS (9). A comparison of the major clinical features of both conditions is shown in **Figure 2**. As the child is currently only 2 years of age, and as both conditions have a wide range of expression, with varying neurodevelopmental trajectories, it is not possible at present to say whether the combination of both conditions will lead to a more severe phenotype in the child as he ages. He is already engaged in a childhood multi-disciplinary early intervention programme, and will have developmental paediatrics, cardiac and endocrine follow up to address the clinical issues seen in both conditions.

This case underscores the diagnostic complexities and phenotypic interplay of two concurrent rare genetic syndromes. Both conditions share overlapping features, including intellectual disability and growth retardation, necessitating comprehensive genetic and clinical evaluation. It also raises the possibility that the presence of a maternal SMC1A may have predisposed to the development of 49,XXXXY through disrupted cohesin regulation of cell division.

## Data Availability

The raw data supporting the conclusions of this article will be made available by the authors, without undue reservation.
